# Supplementary material for: Modeling glioblastoma heterogeneity as a dynamic network of cell states
Source: Mol Syst Biol. 2021 Sep 16;17(9):e10105. doi: 10.15252/msb.202010105 (PMC8444284; doi:10.15252/msb.202010105)
Supplement: Supplementary file 6 — Source Data for Figure 5 [file MSB-17-e10105-s004.zip › Figure5A_sourcedata/GSEA_3071/hallmarks_stateIV.GseaPreranked.1623742551702/HALLMARK_KRAS_SIGNALING_UP.html]

Details for gene set HALLMARK\_KRAS\_SIGNALING\_UP[GSEA]

|  || Dataset | state53071 |
| Phenotype | NoPhenotypeAvailable |
| Upregulated in class | na\_pos |
| GeneSet | HALLMARK\_KRAS\_SIGNALING\_UP |
| Enrichment Score (ES) | 0.27639028 |
| Normalized Enrichment Score (NES) | 1.4154885 |
| Nominal p-value | 0.04368932 |
| FDR q-value | 0.14475146 |
| FWER p-Value | 0.763 |
Table: GSEA Results Summary

  

Fig 1: Enrichment plot: HALLMARK\_KRAS\_SIGNALING\_UP      
 Profile of the Running ES Score & Positions of GeneSet Members on the Rank Ordered List

  

| PROBE | GENE SYMBOL | GENE\_TITLE | RANK IN GENE LIST | RANK METRIC SCORE | RUNNING ES | CORE ENRICHMENT || 1 | MAP3K1 |  |  | 42 | 1.903 | 0.0222 | Yes |
| 2 | IGFBP3 |  |  | 59 | 1.794 | 0.0476 | Yes |
| 3 | PCSK1N |  |  | 110 | 1.507 | 0.0620 | Yes |
| 4 | GPRC5B |  |  | 142 | 1.390 | 0.0782 | Yes |
| 5 | ID2 |  |  | 149 | 1.378 | 0.0989 | Yes |
| 6 | DUSP6 |  |  | 156 | 1.359 | 0.1194 | Yes |
| 7 | ETV5 |  |  | 174 | 1.305 | 0.1368 | Yes |
| 8 | CXCR4 |  |  | 197 | 1.269 | 0.1528 | Yes |
| 9 | SOX9 |  |  | 214 | 1.223 | 0.1692 | Yes |
| 10 | SATB1 |  |  | 222 | 1.203 | 0.1869 | Yes |
| 11 | RGS16 |  |  | 237 | 1.178 | 0.2030 | Yes |
| 12 | KIF5C |  |  | 240 | 1.170 | 0.2212 | Yes |
| 13 | PLAT |  |  | 281 | 1.070 | 0.2305 | Yes |
| 14 | G0S2 |  |  | 370 | 0.942 | 0.2286 | Yes |
| 15 | TRIB2 |  |  | 396 | 0.911 | 0.2383 | Yes |
| 16 | GYPC |  |  | 474 | 0.833 | 0.2368 | Yes |
| 17 | ETV1 |  |  | 488 | 0.824 | 0.2474 | Yes |
| 18 | GNG11 |  |  | 490 | 0.823 | 0.2603 | Yes |
| 19 | HDAC9 |  |  | 510 | 0.810 | 0.2695 | Yes |
| 20 | HKDC1 |  |  | 605 | 0.725 | 0.2630 | Yes |
| 21 | SCG3 |  |  | 632 | 0.707 | 0.2693 | Yes |
| 22 | PRRX1 |  |  | 653 | 0.689 | 0.2764 | Yes |
| 23 | NIN |  |  | 869 | 0.574 | 0.2443 | No |
| 24 | SPRY2 |  |  | 871 | 0.573 | 0.2532 | No |
| 25 | MYCN |  |  | 948 | 0.544 | 0.2473 | No |
| 26 | SCG5 |  |  | 961 | 0.539 | 0.2535 | No |
| 27 | GADD45G |  |  | 1147 | 0.481 | 0.2257 | No |
| 28 | TSPAN7 |  |  | 1181 | 0.471 | 0.2269 | No |
| 29 | MAFB |  |  | 1248 | 0.452 | 0.2214 | No |
| 30 | ST6GAL1 |  |  | 1380 | 0.421 | 0.2030 | No |
| 31 | PEG3 |  |  | 1514 | 0.386 | 0.1836 | No |
| 32 | TMEM158 |  |  | 1588 | 0.369 | 0.1755 | No |
| 33 | HOXD11 |  |  | 1611 | 0.366 | 0.1771 | No |
| 34 | AKT2 |  |  | 1663 | 0.356 | 0.1729 | No |
| 35 | MAP7 |  |  | 1695 | 0.350 | 0.1726 | No |
| 36 | CMKLR1 |  |  | 1705 | 0.349 | 0.1764 | No |
| 37 | ETV4 |  |  | 1937 | 0.307 | 0.1370 | No |
| 38 | SNAP25 |  |  | 2289 | 0.257 | 0.0738 | No |
| 39 | ETS1 |  |  | 2305 | 0.254 | 0.0749 | No |
| 40 | CDADC1 |  |  | 2624 | -0.285 | 0.0185 | No |
| 41 | FBXO4 |  |  | 2906 | -0.319 | -0.0303 | No |
| 42 | LY96 |  |  | 2935 | -0.323 | -0.0305 | No |
| 43 | KLF4 |  |  | 3019 | -0.335 | -0.0411 | No |
| 44 | LCP1 |  |  | 3076 | -0.342 | -0.0464 | No |
| 45 | AMMECR1 |  |  | 3077 | -0.342 | -0.0410 | No |
| 46 | CROT |  |  | 3168 | -0.355 | -0.0526 | No |
| 47 | TNFAIP3 |  |  | 3248 | -0.368 | -0.0619 | No |
| 48 | YRDC |  |  | 3376 | -0.387 | -0.0801 | No |
| 49 | BIRC3 |  |  | 3382 | -0.388 | -0.0749 | No |
| 50 | PSMB8 |  |  | 3446 | -0.398 | -0.0806 | No |
| 51 | PTPRR |  |  | 3466 | -0.402 | -0.0779 | No |
| 52 | ITGB2 |  |  | 3649 | -0.435 | -0.1059 | No |
| 53 | CTSS |  |  | 3693 | -0.443 | -0.1071 | No |
| 54 | PPP1R15A |  |  | 3793 | -0.465 | -0.1187 | No |
| 55 | PRELID3B |  |  | 3870 | -0.480 | -0.1256 | No |
| 56 | HBEGF |  |  | 3905 | -0.488 | -0.1244 | No |
| 57 | DCBLD2 |  |  | 4038 | -0.521 | -0.1414 | No |
| 58 | ITGBL1 |  |  | 4095 | -0.539 | -0.1435 | No |
| 59 | BPGM |  |  | 4213 | -0.570 | -0.1569 | No |
| 60 | GLRX |  |  | 4374 | -0.626 | -0.1776 | No |
| 61 | KCNN4 |  |  | 4421 | -0.642 | -0.1763 | No |
| 62 | ERO1A |  |  | 4434 | -0.647 | -0.1683 | No |
| 63 | EPHB2 |  |  | 4657 | -0.768 | -0.1986 | No |
| 64 | LIF |  |  | 4841 | -0.907 | -0.2193 | No |
| 65 | GFPT2 |  |  | 4843 | -0.910 | -0.2050 | No |
| 66 | TMEM100 |  |  | 4976 | -1.053 | -0.2136 | No |
| 67 | EMP1 |  |  | 4977 | -1.056 | -0.1968 | No |
| 68 | NRP1 |  |  | 5177 | -1.553 | -0.2103 | No |
| 69 | F13A1 |  |  | 5187 | -1.602 | -0.1866 | No |
| 70 | TFPI |  |  | 5212 | -1.705 | -0.1641 | No |
| 71 | PLAUR |  |  | 5213 | -1.721 | -0.1367 | No |
| 72 | ITGA2 |  |  | 5234 | -1.908 | -0.1102 | No |
| 73 | CFH |  |  | 5256 | -2.163 | -0.0799 | No |
| 74 | SPP1 |  |  | 5277 | -2.671 | -0.0413 | No |
| 75 | INHBA |  |  | 5280 | -2.740 | 0.0019 | No |
Table: GSEA details [plain text format]

  

Fig 2: HALLMARK\_KRAS\_SIGNALING\_UP: Random ES distribution      
 Gene set null distribution of ES for **HALLMARK\_KRAS\_SIGNALING\_UP**
